# Impact of COVID-19 Pandemic in a Pediatric and Congenital Cardiovascular Surgery Program in Brazil

**DOI:** 10.21470/1678-9741-2020-0657

**Published:** 2021

**Authors:** Leonardo A. Miana, Valdano Manuel, Luiz Fernando Caneo, Tânia Mara Varejão Strabelli, Elisandra Trevisan Arita, Rosângela Monteiro, Marcelo Biscegli Jatene, Fabio B. Jatene

**Affiliations:** 1 Division of Cardiovascular Surgery, Instituto do Coração (InCor), Hospital das Clínicas, Faculdade de Medicina, Universidade de São Paulo, São Paulo, São Paulo, Brazil.; 2 Infection Control Unit, Instituto do Coração (InCor), Hospital das Clínicas, Faculdade de Medicina, Universidade de São Paulo, São Paulo, São Paulo, Brazil.

**Keywords:** SARS-Cov-2, COVID-19, Heart Defects, Congenital, Coronavirus Infection, Pandemics, Congenital Heart Disease

## Abstract

**Introduction:**

The coronavirus disease 2019 (COVID-19) has negatively impacted healthcare services worldwide. We hypothesized that the pandemic would affect our case mix and mortality. Our objective was to study this impact.

**Methods:**

We retrospectively studied all patients who underwent congenital heart surgeries from March 21^st^ to August 21^st^ in 2019 and 2020 using the institutional electronic database. We compared demographic data, preoperative and postoperative length of stay (LOS), risk stratification using Risk Adjustment for Congenital Heart Surgery (RACHS) classification and outcomes in both periods.

**Results:**

We observed a 66.7% decrease in our surgical volume (285 × 95 patients). Patients operated in the pre-pandemic period were older (911.3 [174.8 - 5953.8] days-old) compared to the pandemic period (275 days-old; *P*<0.05). When the case mix was compared between periods, the percentage of neonatal surgery was increased in the pandemic era (8% × 21.1%; *P*<0.05), and the number of RACHS 1-2 surgeries decreased significantly (60.7 × 27.4%; *P*<0.05). Preoperative LOS was increased in the pandemic period (1.2 × 7 days; *P*=0.001). There was no significant increment in mortality (*P*=0.1). Two patients tested positive for COVID-19 in the postoperative period and both died.

**Conclusion:**

Our program observed a sudden decrease in surgical volume and a consequent increase in surgical complexity. There was a non-significant increment in mortality.

**Table:** 

Abbreviations, acronyms & symbols	
**CHD**	**= Congenital heart disease**
**CI**	**= Confidence interval**
**COVID-19**	**= Coronavirus disease 2019**
**ECMO**	**= Extracorporeal membrane oxygenation**
**ICU**	**= Intensive care unit**
**LOS**	**= Length of stay**
**NA**	**= Non assigned**
**OR**	**= Odds ratio**
**RACHS**	**= Risk Adjustment for Congenital Heart Surgery**
**SARS-CoV-2**	**= Severe acute respiratory syndrome coronavirus 2**

## INTRODUCTION

In March 2020, almost four months after the first severe acute respiratory syndrome coronavirus 2 (SARS-CoV-2) human infection was reported in Wuhan province, China, the first imported case of novel coronavirus disease 2019 (COVID-19) was confirmed in Brazil. At this time, the disease had already shown its complexity around the world, given the high transmission rate (R0 = 1.4-3.8) and a large number of hospitalizations needing intensive care and ventilation support, and exposed the frailty of all the best healthcare systems known^[1-3]^.

COVID-19 has demonstrated a significant impact on healthcare and economics on a scale rarely, if ever, seen in human history^[4]^. This catastrophe imposed an unlimited series of challenges to healthcare systems around the world. These challenges included enormous resource utilization, not only related to equipment and beds but to the human capital and highly specialized medical staff. In addition, there are substantial infection risks to patients, family members, and staff, mainly during the ascending curve of SARS-CoV-2 transmission.

Although there were two initial observational studies reporting infants and children infrequently experiencing a severe clinical manifestation from COVID-19 compared to adults^[5,6]^, soon after the first reported deaths, the Brazilian Ministry of Health recommended postponing or canceling elective surgeries regardless of the pervasiveness of the virus and number of cases in the region and the number and geographical distribution of cases at the time. Pediatric and congenital cardiovascular surgery programs around the globe wondered about how the pandemic would impact or affect their programs^[7]^.

According to initial reports from Wuhan and the Hubei region, patients with cardiovascular comorbidities were at higher risk of morbidity and mortality when associated with COVID-19^[1-3,6]^.

Few data on the impact of COVID-19 in congenital heart disease (CHD) patients were reported as well the impact of this pandemic on those in need of any surgical procedure but still clinically compensated^[8-11]^.

Although the focus of the medical community has shifted toward patients suffering from COVID-19, taking care of CHD patients was still of paramount importance, and it became even more challenging.

The aim of this study was to analyze the impact of the pandemic on the pediatric and adult CHD surgery program at the largest cardiology center in Latin America.

## METHODS

This is a retrospective analysis between March 21^st^ and August 21^st^, 2020 (pandemic period or reduced volume of surgeries) and March 21^st^ and August 21^st^, 2019 (pre-pandemic period or regular volume) using the electronic database of our institution. Routine preoperative tests for COVID-19 started in August; until July, only patients who had symptoms were tested due to the scarcity of tests. The study was conducted at the Instituto do Coração (or InCor) of Universidade de São Paulo, São Paulo, Brazil. The institutional review board and ethics committee approved the study.

For this analysis, we included all patients who underwent congenital heart surgery during these periods comparing their demographic data, preoperative and postoperative length of stay (LOS), age, risk stratification using Risk Adjustment for Congenital Heart Surgery (RACHS) 1, and outcomes^[12,13]^. Patients undergoing heart transplantation were excluded.

The patients were stratified according to their age in:

1. Neonates: < 28 days of life
2. Infants: from 29 days to 12 months old
3. Children: from 12 months to 14 years old
4. Adults: > 14 years old

We used the age of 14 years as a cutoff for adult population because the Brazilian United Health System (or SUS) applies this parameter in Brazilian intensive care units (ICU).

Both groups were compared and predictors for mortality were identified in the entire cohort, specifically studying the pandemic period impact on the outcomes.

### Statistical Analysis

Standard descriptive statistics were calculated. Continuous numerical variables are presented as the median and interquartile range (25^th^ - 75^th^ percentiles). The normality test used was the Kolmogorov-Smirnov test. Chi-square test and Fisher's exact test were used for categorical variables. Survival was estimated using the Kaplan-Meier curve. Mann-Whitney U test was used to compare groups. Binary logistic regression with multivariable analysis was used to identify risk factors for in-hospital mortality. A *P*-value ≤ 0.05 was considered significant. For statistical analysis, we used the softwares IBM Corp. Released 2012, IBM SPSS Statistics for Windows, version 21.0, Armonk, NY: IBM Corp and GraphPad Prism 5 software (San Diego, California, United States of America).

## RESULTS

During the pandemic, we observed a 66.7% decrease in patient volume. Between March 21^st^ and August 21^st^, 2020, we performed 106 surgeries on 95 patients. During the same time span in 2019, we operated 294 surgeries on 285 patients.

**Fig. 1:**
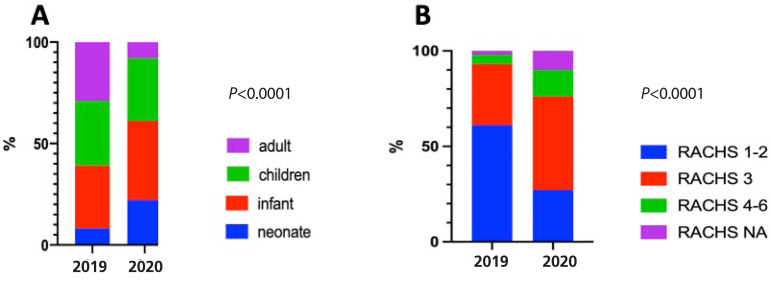
Distribution of the patients according to their age (A) and severity (B). NA=non assigned; RACHS=Risk Adjustment for Congenital Heart Surgery

We also observed a complete change in patient profile and case mix (Table 1; Figure 1A and 1B). The median age of the patients dropped from 911 to 275 days old, with a 260% increase in neonatal surgery (*P*<0.0001; Figure 1A). RACHS distribution exhibited a significant decrease in more simple procedures (RACHS 1 and 2), from 60.7% to 27.4% (*P*<0.0001; Table 1 and Figure 1B).

**Table 1:** Baseline characteristics of the 380 patients analyzed during pre-pandemic and pandemic periods.

Variables	Pre-pandemic period, 2019 (285 patients)	Pandemic period, 2020 (95 patients)	*P*-value
Age (months)	911.3 (174.8-5953,8)	275.4 (88.5-1483)	< 0.0001
Age stratification			< 0.0001
Neonates (0-28 days)	23 (8.1%)	20 (21.1%)	
Infants (1-12 months)	88 (30.9%)	37 (38.9%)	
Children (1-14 years)	92 (32.3%)	30 (31.6%)	
Adults (> 14 years)	82 (28.8%)	8 (8.4%)	
Gender			0.72
Female	144 (50.5%)	46 (48.4%)	
Male	141 (49.5%)	49 (51.6%)	
RACHS			0.0001
1	68 (23.9%)	6 (6.3%)	
2	105 (36.8%)	20 (21%)	
3	91 (32%)	47 (49.5%)	
4	11 (3.9%)	10 (10.5%)	
05/jun	5 (1.7%)	3 (3.2%)	
Non assigned	5 (1.7%)	9 (9.5%)	
Reoperation	62 (21.7%)	17 (17.9%)	0.10
Surgical reinterventions	9 (2.8%)	11 (11.6%)	0.003
Preoperative LOS	1.2 (0.9-4.3)	6.8 (2.8-11.8)	0.0001

LOS=length of stay; RACHS=Risk Adjustment for Congenital Heart Surgery

There were seven patients with endocarditis in the pandemic period (7.4%) with two deaths (28.5%); meanwhile, the incidence in the same period in 2019 was 0.7% (two patients) (*P*=0.001).

There was no significant difference in reoperations (reapproach to upsizing of a conduit, valve repair or replacement, next stage of single-ventricle palliation, or total repair after palliation) as an indication for elective surgery when comparing the periods (21.7% × 17.9%; *P*=0.1), but there was a significant increase in the surgical interventions during the same hospitalization (2.8% × 11.6%; *P*=0.003; Table 1) and there is a higher percentage of patients that required surgical reintervention in the same hospitalization during the pandemic period compared to the year before.

Accordingly, during COVID-19 pandemic, it was observed an increased number of days in the hospital before surgery (1.2 × 6.8 days, *P*<0.001; Table 1).

### Outcomes

We observed a 66.7% decreased in the total volume of pediatric and congenital cardiovascular surgeries during the pandemic period compared to the same period in 2019.

Due to the relatively increased number of neonates and complex surgeries, the median days at postoperative ICU was longer during the pandemic period - 6.8 days compared to 4.1 days between March 21^st^ and August 21^st^, 2019 (*P*=0.02; Table 2). Total hospital LOS was also affected (13.4 × 22.6; *P*<0.001; Table 2).

**Table 2:** Surgical outcomes.

Variables	Pre-pandemic period, 2019 (285 patients)	Pandemic period, 2020 (95 patients)	*P*-value
ICU LOS	4.1 (2.1-13.4)	6.8 (3.3-12.8)	0.02
Total hospital LOS	13.4 (8.2-27.2)	22.6 (15.1-34.1)	0.0001
30-day mortality	17 (6%)	10 (10.5%)	0.13
Overall mortality	23 (8.1%)	13 (13.7%)	0.10

ICU=intensive care unit; LOS=length of stay

On the other hand, there was no statistically significant increment in 30-day and overall mortalities in 2020 (*P*=0.1; Table 2).

Both univariate and multivariate analyses identified neonatal surgery (*P*<0.001) and RACHS-1 category (*P*<0.001) and not the pandemic period as predictors for mortality (Table 3).

**Table 3:** Univariate and multivariate risk factors for mortality.

Variable	Univariate			Multivariate		
	OR	95% CI	*P*-value	OR	95% CI	*P*-value
Age						
Sex	1.6	0.8-3.3	0.16			
Reoperation	0.9	0.4-2.1	0.8			
Need for reintervention	1.4	0.3-6.3	0.6			
RACHS ≥3	10.3	3.5-29.7	<0.0001	6.4	2.1-19.7	0.001
Pandemic period	1.8	0.8-3.7	0.11	1.2	0.4-1.9	0.7
Neonatal surgery	10.9	5.1-23.6	<0.0001	6.0	2.7-13.6	<0.0001

## DISCUSSION

Brazil was one of the most affected countries by the COVID-19 pandemic with more than 8.318/million serious or critical cases needing hospitalization and ICU care^[14]^.

The Brazilian healthcare infrastructure faced unprecedented challenges with the COVID-19 pandemic, and the CHD community was no exception. Patients avoided seeking medical attention for fear of catching the deadly condition or as an unintended consequence of *stay-at-home* orders. This delay in seeking medical care has drastically decreased emergency room visits for non-COVID conditions such as appendicitis, heart attack, stroke, and other diseases, including CHD.

Elective surgeries were postponed or canceled, including those for CHD. It was not only a local situation, but a global recommendation^[7]^.

In this retrospective analysis of the largest Brazilian program of congenital heart surgery, we have observed a 66.7% decrease in surgical volume in the pandemic period. As we paused elective cases, there was a relative increase in the complexity of the cases (neonates and higher RACHS-1 classification). The number of RACHS-1 categories 1 and 2 and patients older than one year of age dropped dramatically.

This shift in the case mix is explained by performing mainly urgent and emergency surgeries. This relative increment in neonatal surgery during the pandemic was observed in other centers and countries^[9,15]^.

The impact of the pandemic on congenital heart surgery programs was not only observed in countries considered epicenters of the pandemic, it was a global problem and one of the COVID-19 pandemic consequences^[10]^.

We have also noticed a peak incidence of bacterial endocarditis as an indication for surgery (seven out of 95 patients [7.4%]). This was observed in six patients in the postoperative period and in one patient with no previous intervention. In this patient without a previous diagnosis of any heart disease, the endocarditis was in the tricuspid valve due to a central venous catheter. This observation is yet to be understood, specifically if there was any relationship with the pandemic period. Due to the risk and fear of contamination by the COVID-19 virus, several patients probably stayed at home with some other types of infection (*i.e*., systemic or dental caries) that were not treated and developed endocarditis during this period, which may explain this increase. The infectious agent was isolated in six patients and five different species were identified.

Institutional protocols have been established to prevent infection of patients both in the pre and postoperative periods. In our hospital, the protocol was regularly revised in order to comply with the most current recommendations and address any issues that arose as the pandemic evolved. In the first five months, between March 21^st^ and August 21^st^, 2020, patients from our general hospital were referred to our cardiac center; we were not able to test every patient and did not have separated patient flows around the hospital. Additionally, in 2020, this was the period with the highest incidence of new infections in our country. ICUs were overloaded in every hospital, including in our unit.

In this period, we had two patients that tested positive for SARS-Cov-2. One of them was an adult referred to our hospital with pulmonary valve endocarditis and mitral regurgitation. She had a preoperative COVID-19 negative test and underwent surgery with unremarkable recovery until the 8^th^ postoperative day, when she was in the ward and an important pericardial effusion was identified. Immediately, pericardial drainage was performed; the patient was extubated and sent back to the ICU. On the same day, she presented with respiratory failure and cardiac arrest. After three minutes of cardiopulmonary resuscitation and reintubation, she recovered the cardiac function and further exams rolled out cardiac tamponade but revealed a severe bilateral pulmonary infiltration. The nasal swab confirmed COVID-19 and she was referred to our general hospital. Her respiratory status improved, and she was able to be extubated; however, while weaning her supplementary nasal cannula oxygen, she began to deteriorate clinically; she had become septic, which ultimately resulted in the patient’s death on the 24^th^ postoperative day.

The other postoperative COVID-19 patient was an 11-month-old child with tricuspid atresia/pulmonary stenosis but no previous intervention. He presented in our emergency room with severe hypoxemia. There were no signs of pulmonary infiltration on the admission X-ray and room air saturation was 50%. An echocardiogram revealed a severely restrictive atrial and ventricular septal defect. An urgent Glenn procedure with atrial septal defect enlargement was performed. The patient had postoperative bleeding and needed chest re-exploration, but then presented with good recovery and was extubated. Nevertheless, hypoxemia resumed, and an X-ray revealed severe bilateral pulmonary infiltration. Nasal swab confirmed COVID-19 infection. Hypoxemia was not responsive to intubation and mechanical ventilation measurements. Although Glenn anastomosis was patent and with good flow, his oxygen saturations remained in the 60%. On the 5^th^ postoperative day, he had three resuscitated short cardiac arrests due to profound cyanosis followed by cardiac decompensation and was successfully resuscitated. The patient was cannulated on venous-venous extracorporeal membrane oxygenation (ECMO). He showed signs of pulmonary image and function healing, but on the 20^th^ day of ECMO run, a fungal sepsis refractory to antimicrobial treatment was identified. The patient was weaned off ECMO but died on the 30^th^ postoperative day.

Since August 21^st^, 2020, our institutional protocol consists of dividing the hospital and the operative theatre into two parts - one called COVID-free area, for elective cases, patients recently tested negative for SARS-CoV-2 infection, and the other being a risk zone for patients with urgent and emergency operations and suspected or positive COVID-19 test.

Different protocols and guidelines have been proposed around the world with the aim to establish a perioperative management issue for patients with CHD^[9,10,16,17]^.

We have not observed a statistically significant rise in global postoperative mortality. Both univariate and multivariate analyses identified neonatal surgery and higher RACHS category as independent risk factors. Since we had relatively more neonatal and complex cases in this period, the non-statistically significant higher mortality can be easily understood - and added to this is the fact that we lost two additional patients due to COVID-19 infection.

The pandemic impacted the surgical volume and case mix, but based on our findings, it was not a risk factor for morbidity or mortality.

Unfortunately, the two patients who were infected with COVID-19 died, highlighting the severity of the disease, specifically in the immunosuppressed postoperative period.

With increasingly safer protocols and guidelines in times of pandemic, surgical outcomes for CHD can be very close to those of the pre-COVID-19 era.

### Limitations

The main limitation is the fact that it is a retrospective single-center study.

## CONCLUSION

In conclusion, COVID-19 had a severe impact on our Pediatric and Congenital Cardiac Surgery Program with a 66.7% decrease in surgical volume, increasing the relative incidence of complex surgical cases. There was no statistically significant rise in mortality compared to the same period in 2019.

**Table:** 

Authors' roles & responsibilities	
LAM	Substantial contributions to the conception and design of the work; and the analysis of data for the work; drafting the work and revising it critically for important intellectual content; final approval of the version to be published
VM	Substantial contributions to the conception and design of the work; drafting the work and revising it critically for important intellectual content; final approval of the version to be published
LFC	Substantial contributions to the conception and design of the work; drafting the work; final approval of the version to be published
TMVS	Substantial contributions to the acquisition of data for the work; final approval of the version to be published
ETA	Substantial contributions to the acquisition of data for the work; final approval of the version to be published
RM	Substantial contributions to the acquisition of data for the work; final approval of the version to be published
MBJ	Substantial contributions to the conception and design of the work; drafting the work and revising it critically for important intellectual content; final approval of the version to be published
FBJ	Substantial contributions to the conception and design of the work; drafting the work and revising it critically for important intellectual content; final approval of the version to be published
